# Comparative research of intestinal microbiota diversity and body mass regulation in *Eothenomys miletus* from different areas of Hengduan mountain regions

**DOI:** 10.3389/fmicb.2022.1026841

**Published:** 2022-10-17

**Authors:** Bowen Yan, Ting Jia, Zhengkun Wang, Wanlong Zhu

**Affiliations:** ^1^Key Laboratory of Ecological Adaptive Evolution and Conservation on Animals-Plants in Southwest Mountain Ecosystem of Yunnan Province Higher Institutes College, School of Life Sciences, Yunnan Normal University, Kunming, China; ^2^Yunnan College of Business Management, Kunming, China; ^3^Engineering Research Center of Sustainable Development and Utilization of Biomass Energy, Ministry of Education, Kunming, China; ^4^Key Laboratory of Yunnan Province for Biomass Energy and Environment Biotechnology, Kunming, China

**Keywords:** *Eothenomys miletus*, Hengduan mountain region, gut microbes, body mass regulation, adaptation

## Abstract

In order to investigate the effects of different areas on intestinal bacterial diversity and body mass regulation in *Eothenomys miletus* from Hengduan mountain regions, and to explore the community structure and diversity of intestinal microflora and their role in body mass regulation. *E. miletus* was collected from five areas including Deqin (DQ), Xianggelila (XGLL), Lijiang (LJ), Jianchuan (JC), and Dali (DL), we used 16S rRNA sequencing technology combined with physiological and morphological methods to study the intestinal microbiota diversity, abundance and community structure of the intestinal bacteria in winter, and to explore the influence of geographical factors, physiological indicators including food intake, resting metabolic rate (RMR), non-shivering thermogenesis (NST), neuropeptide Y (NPY), Agouti-Related Protein (AgRP), proopiomelanocortin (POMC), cocaine and amphetamine regulated transcription peptide (CART), and morphological indicators including body mass, body length and other nine indicators on the intestinal microflora diversity in *E. miletus*. The results showed that there were significant differences in metabolic indexes such as RMR, NST, NPY, AgRP, and morphological indexes such as body length, tail length and ear length among the five regions. Bacterial community in intestinal tract of *E. miletus* mainly includes three phyla, of which Firmicutes is the dominant phyla, followed by Bacteroidetes and Tenericutes. At the genus level, the dominant bacterial genera were S24-7(UG), Clostridiales (UG), and Lachnospiraceae (UG), etc. α diversity of intestinal microorganisms in DL and JC were significantly different from that in the other three regions. Genera of intestinal microorganisms in DL and JC were also the most. Moreover, *Bacteroides*, *Ruminococcus*, and *Treponema* could affect energy metabolism in *E. miletus*, which were closely related to the environment in which they lived. All of these results indicated that different areas in Hengduan Mountain had certain effects on the structure of intestinal microbial community in *E. miletus*, which were responded positively to changes in food abundance and other environmental factors. Furthermore, Firmicutes and Bacteroidetes play an important role in the body mass regulation in *E. miletus*.

## Introduction

Intestinal flora is closely related to the life activities of the host, which co-evolve with the evolution of the host (Bäckhed et al., 2004). Mammalian gut microbes colonize the body from birth and gradually form a relatively stable microbial community when the host maturates, these microbial communities play key roles in host’s physiological and biochemical functions, including immune regulation, food digestion or nutrient absorption (Hooper et al., 2002; Round and Mazmanian, 2009; Lifeng et al., 2011). For example, intestinal microorganisms can stimulate the cellular and humoral immunity of mammals, enhance their immunity, reduce the probability of disease occurrence, and maintain the physiological health of the host (Gaoxue et al., 2010; Nayak, 2010; Chunhung et al., 2012; Kai et al., 2017). Intestinal microorganisms can also enhance the host’s ability to digest and absorb plants with high fiber and low protein, lead to enhance the food utilization rate and tolerance to extreme environments, and maintain the normal physiological activities of animals when food resource is scarce (Montllor et al., 2002; Tsuchida et al., 2004; Kohl et al., 2017). Gut microbes in mammals are affected by various factors, such as habitat change, lifestyle, diet, social activities or environment (David et al., 2003; Amato et al., 2013; Grieneisen et al., 2017; McKenzie et al., 2017; Rothschild et al., 2018), among which environment is an important factor. For example, previously study found that geographical isolation has a great impact on the gut microbiota of *Amblyrhynchus cristatus*, *Macrotus californicus*, and *Pyrrhocoris apterus*, and the structure of gut microbiota of the same species varies significantly under different geographical conditions (Lankau et al., 2012; Phillips et al., 2012; Sudakaran et al., 2012). Studies on *Macaca mulatta* in different regions of China have found that the intestinal microorganisms of *M. mulatta* in Beijing and Henan are not as rich as those in Guangxi and Fujian (Li et al., 2016). Studies on *Mus musculus* have found that the feeding environment has a great influence on the structure of intestinal microbiota, while the influence of gender difference is relatively small (Yeshi and Xin, 2012). Therefore, studying the changes of the gut microbial community of the same species under different environments can better understand the adaptability of the species to different environments.

Environmental differences will not only affect the intestinal microbial changes of animals, but also affect their population life strategies; the same species may form different life strategies under different environments (Poblete et al., 2018). Animals can adapt to their environment by changing their size, personality or physiological characteristics, such as body size in mammals, birds and amphibians increases with the increasing of latitude, while the body size of reptiles decreases with the increasing of latitude (Ashton and Feldman, 2003; Ashton, 2004). Physiological regulation of body mass and energy metabolism are the main strategy for small mammals to cope with environmental changes, which are also of great significance for enhancing their adaptive ability and improving their survival chances (Heldmaier et al., 1982; Xueying and Dehua, 2006; Dehua, 2011). For example, studies on *Eospalax cansus* have found that the temperature and humidity in different regions have significant effects on its body mass (Shicai et al., 2017). For *Lasiopodomys brandtii*, *Microtus oeconomus*, *Ochotona curzoniae*, *Microtus pennsylvanicus*, and *Phodopus sungorus*, it was also found that their body mass and energy metabolism would change with the changing of environment (Iverson and Turner, 1974; Mercer, 1998; Xingsheng and Dehua, 2005; Jianmei et al., 2006a,b). Previous studies in our laboratory have also found that body mass and energy metabolism in *Eothenomys miletus* was also changed significantly with the different living environment (Chunyan et al., 2021). Differences in gut microbes of animals in different regions may also affect the physiology or morphology of the animals, and ultimately affect the survival of the species.

Hengduan mountain regions are located in the interchange between Palaearctic and Oriental, which is a unique mountain valley in China, as one of the global biodiversity hotspots (Zhengda et al., 2001). With the intense of altitude change, its climate changes are diverse, which showed seasonal variation of vegetation resources, indicating that there are obvious differences in the physiological and ecological characteristics of small mammals in different regions (Wanlong et al., 2010). *Eothenomys miletus* belongs to the genus *Eothenomys* in Arvicolinae, which is a endemic species in Hengduan mountain regions of China. *E. miletus* is mostly active at night and feeds on fresh serosa plants and grass roots (Zwxun et al., 2000). Our previous studies on the adaptability in *E. miletus* mainly focused on physiological and morphological aspects in winter (Wanlong et al., 2009; Haiji et al., 2018), as well as the changes in body mass regulation under different food quantities or qualities (Wenrong et al., 2013; Xuena et al., 2021). However, the important role of intestinal flora in their life activities was not considered. Based on the 16S rRNA gene high-throughput sequencing technology, combined with relevant physiological and morphological indicators, the present study investigated the differences in gut microbes and body mass regulation in *E. miletus* of different areas from Hengduan mountain region in winter, analyzed the changes in intestinal bacterial diversity and explored the response mechanism of intestinal bacteria to body mass regulation of *E. miletus*. We hypothesized that the intestinal microbial diversity in different regions would be affected by their altitude or plant types in *E. miletus*. We predicted that there were regional differences in intestinal microbial diversity, and the composition of their intestinal flora may affect the physiology or morphology, and ultimately affect their body mass regulation in *E. miletus.*

## Materials and methods

### Sample collection

*Eothenomys miletus* were captured in Deqin (DQ), Xianggelila (XGLL), Lijiang (LJ), Jianchuan (JC), and Dali (DL) in winter of 2020. Animals were healthy adult individuals in the non-reproductive period. All animal procedures were within the rules of Animals Care and Use Committee of School of Life Sciences, Yunnan Normal University. This study was approved by the Committee (13-0901-011). The geographic location, climatic characteristics, and sample size of each sample point were detailed in Table 1. The climatic data used in the analysis was the data of year 2020 and are downloaded from the National Meteorological Science Data Center^1^ for each sampling site.

**TABLE 1:** Geographical locations and main conditions for five populations of *Eothenomys miletus.*

Regions	Sample number	Longitude and latitude	Altitude/m	Mean winter temperature/°C	Precipitation/mm	Vegetation type
DQ	9 (5♂/4♀)	99°03′75″ E, 28°35′14″ N	3459	3.0	633.7	Alpine meadows
XGLL	10 (7♂/3♀)	99°83′16″ E, 27°90′73″ N	3321	4.5	984.2	Subalpine meadows
LJ	10 (6♂/4♀)	100°22′90″ E, 26°87′53″ N	2478	10.5	975.0	Subalpine meadows and shrublands
JC	11 (8♂/3♀)	99°75′03″ E, 26°43′95″ N	2590	11.0	987.3	Leafy bushes
DL	10 (7♂/3♀)	100°42′49″ E, 24°90′30″ N	2217	17.5	597.0	Savannah shrubs

DQ, Deqin; XGLL, Xianggelila; LJ, Lijiang; JC, Jianchuan; DL, Dali.

### Measurement of physiological indicators

After capture, resting metabolic rate (RMR), non-shivering thermogenesis (NST) and food intake were measured in the field, and the individuals tested before RMR and NST were measured fasted for 2–3 h and left in the respiratory chamber for 0.5 h. Portable breathing apparatus (FMS-1901-03, USA) is used for measurement, food intake was measured by the food balance method, and the specific measurement method is described in Zhu et al. (Wanlong et al., 2008). Then serum, rectal stool and hypothalamus were taken, serum leptin concentration and hypothalamic neuropeptide gene expression were determined by radioimmunoassay and real-time fluorescence quantification, respectively, as detailed in Wanlong et al. (2017).

### Measurement of morphological indicators

Morphological indicators were measured with reference to the methods of Yang et al. and Xia et al. (Qisen et al., 2005; Lin et al., 2006): body mass (accurate to 0.01 g), body length, tail length, ear length, ear width, forelimb length, hindlimb length, cranial length, and cranial base length, upper tooth row length, lower tooth row length (accurate to 0.01 cm).

### DNA extraction

Take 0.1 g of rectal stool, total DNA enriched on the membrane was extracted using the Centrifugal Column Soil Genome Extraction Kit (DNeasy^®^ PowerSoil^®^ Kit, Germany).

### High-throughput sequencing

The purified PCR product was determined using a Nanodrop 2000 spectrophotometer, where nucleic acid concentrations above 10 ng/uL and purity (A260/A180) greater than 1.8 were valid samples. Molars such as DNA samples with qualified concentrations after purification were mixed and sequenced using the Illumina Miseq platform (Illumina, San Diego, CA, USA).

### Bioinformatics analysis

A double-terminal sequence of 2 × 250 bp is obtained by sequencing on the Illumina Miseq platform, and the processing and analysis of these raw data is processed and analyzed using the QIIME platform. The double-end sequence is first stitched using Flash software, and then each sample is matched with a unique barcode label. Remove low-mass sequences (sequence length less than 300 or base mass fraction less than 30) during stitching. Use the Usearch 7.0 software to remove chimeras from the sequence, and then cluster all sequences with 97% similarity as operational taxonomic unit (OTU) by the Ulust algorithm. Select the sequence with the longest sequence length as the representative sequence and use the Ribosomal Database Project database to annotate the sequence classification information. Finally, to compare all the samples, we normalized the sequences of all the samples through the “Daisychopper” script code, each with a standard of 5,437 sequences.

### Statistical analysis

#### Microbial community composition

Create a percentage stacked bar chart using Origin 2018 to describe bacterial communities.

#### α, β diversity

α Diversity is estimated through 2 diversity indicators: Chao 1 and shannon diversity, and described by the creation of box graphs through Origin 2018. β diversity: the One-way ANOVA and Tukey post-mortem were used in SPSS 26.0 to see if differences in diversity between the two groups were significant, and community structures were described using an unweighted and weighted UniFrac distance matrix using QIIME and Origin 2018. Unweighted UniFrac distance depends on phylogenetic relationships and OTU species abundance, while species deletion/presence and phylogenetic relationships are considered by weighted UniFrac.

#### Venn diagrams

Common and unique parameters between Venn diagrams analyzed online in Venn 2.1.^2^

#### Enrichment analysis

Uses a one-way ANOVA method.

#### Heat map of environmental physicochemical properties in different regions associated with dominant microorganisms in velvet manure

Pearson analysis using SPSS 26.0 and R 3.6.2 obtain the relevant heat map.

#### RDA analysis

Using Canoco 5.0, redundancy analysis (RDA) was used to assess the correlation between dominant genera (top 9) and physicochemical factors.

#### Network analysis

These results were further analyzed using R 3.6.2 and Gephi v.0.9.2 software to generate network analysis (*P* < 0.05, |r| > 0.6).

### Physiological and morphological index analysis

Data were analyzed using SPSS 26.0 software (SPSS Inc., Chicago, IL, USA). Before all statistical analyses, data were examined for normality and homogeneity of variance using Kolmogorov–Smirnov and Levene tests, respectively. Differences in physiological indicators between the different sexes of *E. miletus* in the same region were not significant, so all data were combined and counted. Regional difference in body mass was analyzed by One-way ANOVA. Regional differences in food intake, expression of neuropeptide in hypothalamus, body length and other nine morphological indicators of five regions were analyzed by One-way covariance analysis (ANCOVA), using body mass as a covariate. Results are expressed in mean ± SE, *P* < 0.05 was significantly different.

## Results

### Influence of region on intestinal microorganisms

**FIGURE 1:**
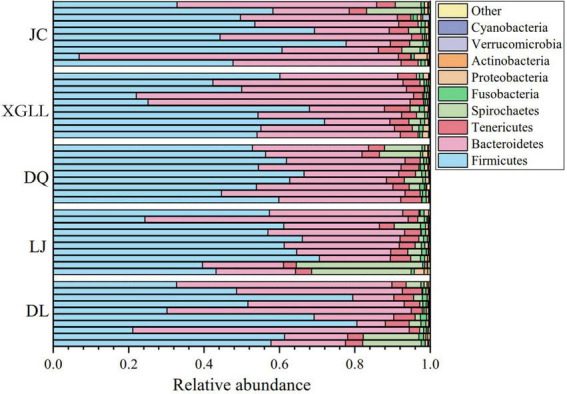
Microbial phylum horizontal community composition in *Eothenomys miletus.*

**FIGURE 2:**
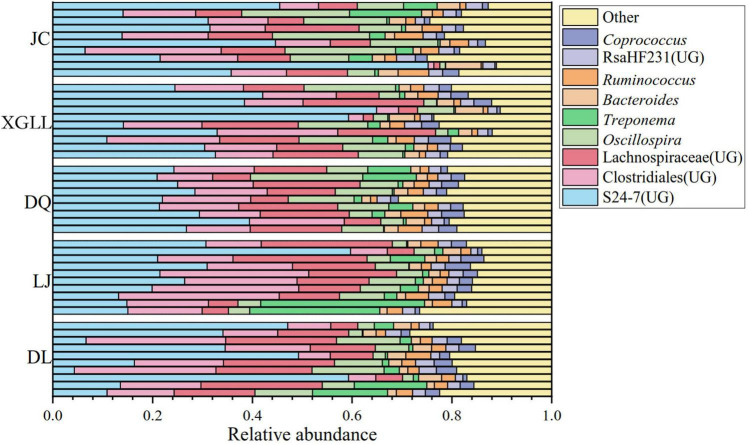
Microbial genera level microbial composition in *Eothenomys miletus.*

A total of 50 samples were collected for DNA extraction and PCR product amplification, and each sample was normalized to 5,437 sequences after removing low-quality sequences, chimeras, monomers, and chloroplasts. We also analyzed the composition of the gut microbiota at the phylum or genus level. According to the classification results based on OUT, at the phylum level, three phylum were the dominant microorganisms in the feces of large chutes in the five regions (Figure 1), which were Firmicutes (52.95%), Bacteroidetes (36.06%), and Tenericutes (4.45%). At the genus level, three genera dominated the dominant microorganisms in the feces of large chorionic rodents in the five regions (Figure 2), which were: S24-7 (UG) (29.32%), Clostridiales (UG) (15.25%), and Lachnospiraceae (UG) (13.44%).

### Differences in intestinal microbial α and β diversity of different regions

**FIGURE 3:**
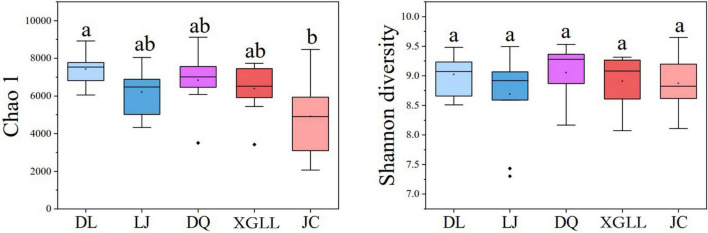
α diversity of feces in *Eothenomys miletus* in different regions.

**FIGURE 4:**
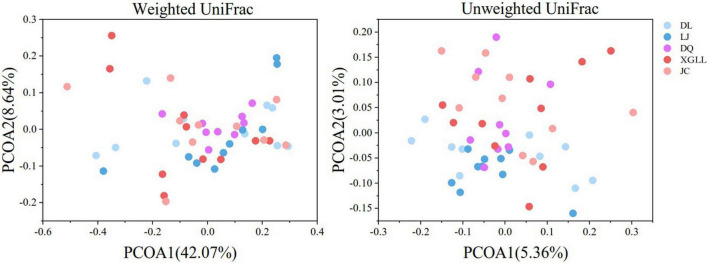
β diversity of feces in *Eothenomys miletus* in different regions.

Alpha (α) diversity (Chao1 and Shannon diversity) of fecal microorganisms from all regions was showed in Figure 3. Among them, only DL and JC showed significant difference in Chao1 index (*P* < 0.05), while no significant difference was found in other areas (*P* > 0.05). These results indicated that intestinal microbial diversity in DL was significantly higher than that in LJ, DQ, and XGLL, while the intestinal microbial diversity in JC was significantly lower than that in these three areas. There was no significant difference in Shannon diversity of fecal microorganisms in all regions (*P* > 0.05), which indicated that there is no significant difference in the evenness of intestinal bacterial flora in these five regions. As for the beta (β) diversity (weighted UniFrac distance) of fecal microbiota from different regions, we found that the distribution of β diversity was not significantly different (*F* = 1.118; *R*^2^ = 0.092; *P* = 0.308, Figure 4). However, based on the unweighted UniFrac distance, we found there is a significant difference among different areas (*F* = 1.067; *R*^2^ = 0.088; *P* = 0.020).

### Distribution of common and endemic microorganisms in different areas

**FIGURE 5:**
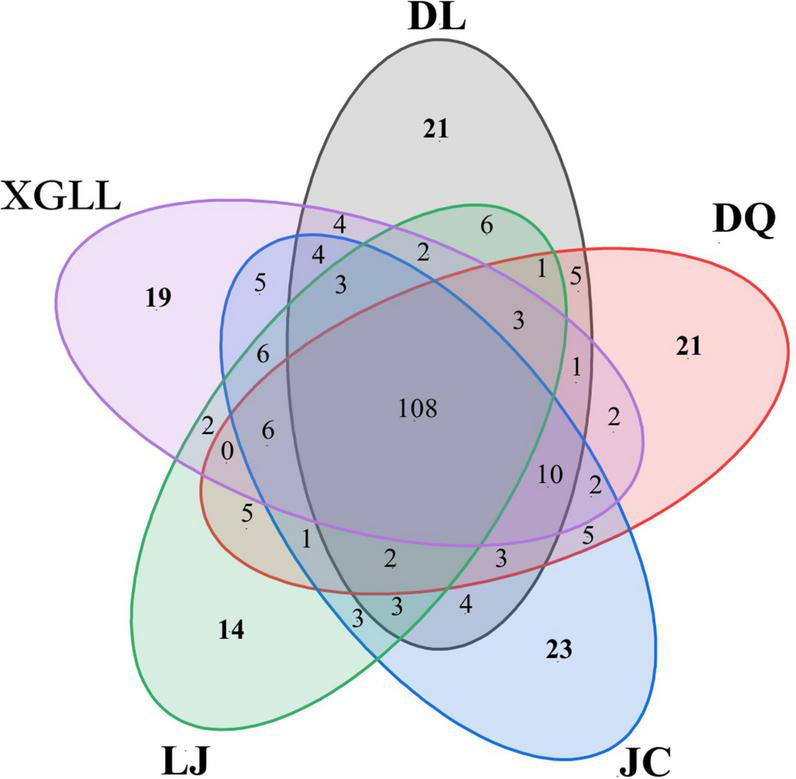
Venn diagram of fecal microbials of *Eothenomys miletus* in different regions.

Venn diagrams can be intuitive show total of each sample and unique microbial quantity and proportion, through the analysis found that the number of microorganisms of the genus in JC and DL, most of 188 and 180, respectively. XGLL and DQ for 177 and 175, LJ prefecture of microbial quantity minimum, only for 165 kinds (Figure 5). A total of 108 genera were found in the fecal microbiota in different areas, accounting for 36.73% of the total. Among them, 21 genera were endemic to DL, 14 genera were endemic to LJ, 21 genera were endemic to DQ, 19 genera were endemic to XGLL, and 23 genera were endemic to JC. XGLL and JC had the highest similarity of intestinal microbiota genera (65.16%), while LJ and DQ had the lowest similarity (58.88%). These results indicate that there was not only common microbiota but also unique microbiota in the gut of *E. miletus* in different regions.

### Enrichment analysis of different microorganisms in different regions

**FIGURE 6:**
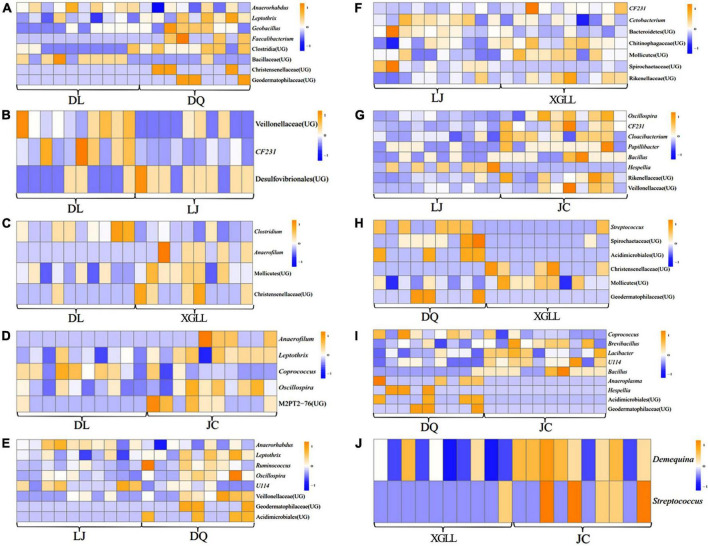
Differential microbial analyses of feces in *Eothenomys miletus* between the two regions. **(A)** DL vs. DQ; **(B)** Dl vs. LJ; **(C)** DL vs. XL; **(D)** DL vs. JC; **(E)** LJ vs. DQ; **(F)** LJ vs. XL; **(G)** LJ vs. JC; **(H)** DQ vs. XL; **(I)** DQ vs. JC; **(J)** XL vs. JC.

Compared with DQ, *Faecalibacterium* and Clostridia (UG) in DL were significantly enriched in the feces of *E. miletus* (*P* < 0.05, Figure 6A). Compared with DL, Veillonellaceae (UG), and CF231 were significantly enriched in LJ (*P* < 0.05, Figure 6B). Compared with XGLL, *Anaerofilum* and Christensenellaceae (UG) were significantly enriched in DL (*P* < 0.05, Figure 6C). Compared with JC, *Anaerofilum* and M2PT2-76 (UG) were significantly enriched in DL (*P* < 0.05, Figure 6D). Compared with DQ, Geodermatophilaceae (UG) and Acidimicrobiales (UG) were significantly enriched in LJ (*P* < 0.05, Figure 6E). Compared with XGLL, *CF231* and Rikenellaceae (UG) were significantly enriched in LJ (*P* < 0.05, Figure 6F). Compared with JC, *Oscillospira*, *CF231* and Cloacibacterium were significantly enriched in LJ (*P* < 0.05, Figure 6G). Compared with DQ, *Streptococcus* and Spirochaetaceae (UG) were significantly enriched in XGLL (*P* < 0.05, Figure 6H). Compared with DQ, *Anaeroplasma* and *Hespellia* were significantly enriched in JC (*P* < 0.05, Figure 6I). Compared with JC, *Demequina* and *Streptococcus* were significantly enriched in XGLL (*P* < 0.05, Figure 6J).

### Relationship between environmental factors, physiological indexes, morphological indexes, and intestinal microorganisms in different regions

Results of One-way ANOVA or ANCOVA showed that among the morphological indexes (Table 2), body mass, body length, tail length, forelimb length, and cranial base length in *E. miletus* among DL, LJ and JC were significantly higher than those in DQ and XGLL. Ear length was larger in DL and XGLL, smaller in DQ and JC. Ear width and cranial base length were larger in DL, but smaller in XGLL. Length of the upper teeth is longer in JC and shorter in DQ. In terms of physiological indicators (Table 3), food intake, RMR, NST, and NPY and AgRP expressions were significantly higher in DQ and XGLL than in DL, LJ, and JC. Serum leptin was the highest in DL, and the lowest in DQ and XGLL.

**TABLE 2:** Comparison of morphological indicators in *Eothenomys miletus.*

Trait	Regions					*F*	*P*
	DL	LJ	DQ	XGLL	JC		
BM	40.600 ± 0.922^a^	35.851 ± 0.985^b^	30.360 ± 2.278^c^	31.943 ± 1.348^c^	41.286 ± 1.089^a^	13.093	< 0.01
BL	15.605 ± 0.050^a,b^	15.467 ± 0.109^a,b^	14.081 ± 0.210^c^	15.210 ± 0.248^b^	15.828 ± 0.054^a^	12.002	< 0.01
TL	4.206 ± 0.025^a^	4.066 ± 0.041^a,b^	3.694 ± 0.026^c^	4.020 ± 0.091^b^	4.189 ± 0.059^a^	6.758	< 0.01
EL	1.267 ± 0.012^a^	1.210 ± 0.019^a,b^	1.132 ± 0.138^c^	1.277 ± 0.044^a^	1.161 ± 0.021^b,c^	6.149	< 0.01
EW	1.309 ± 0.018^a^	1.258 ± 0.024^a,b^	1.261 ± 0.021^a,b^	1.184 ± 0.014^c^	1.247 ± 0.010^b^	4.803	< 0.01
FLL	2.578 ± 0.043^b^	2.799 ± 0.459^a^	2.372 ± 0.037^c^	2.320 ± 0.043^c^	2.746 ± 0.022^a^	20.573	< 0.01
HLL	3.605 ± 0.098	3.505 ± 0.256	3.546 ± 0.096	3.547 ± 0.248	3.669 ± 0.136	0.780	> 0.05
CL	2.645 ± 0.010^a^	2.579 ± 0.017^b^	2.557 ± 0.008^b,c^	2.515 ± 0.035^c^	2.572 ± 0.013^b,c^	4.085	< 0.01
CBL	2.588 ± 0.006^a^	2.479 ± 0.015^b,c^	2.438 ± 0.007^c,d^	2.422 ± 0.028^d^	2.512 ± 0.019^b^	8.335	< 0.01
UTRL	1.604 ± 0.008^a,b^	1.601 ± 0.004^a,b^	1.551 ± 0.009^c^	1.579 ± 0.022^b,c^	1.629 ± 0.003^a^	4.332	< 0.01
LTRL	1.158 ± 0.015	1.148 ± 0.027	1.162 ± 0.027	1.124 ± 0.072	1.153 ± 0.017	1.604	> 0.05

BM, body mass; BL, body length; TL, tail length; EL, ear length; EW, ear width; FLL, forelimb length; HLL, hindlimb length; CL, cranial length; CBL, cranial base length; UTRL, upper tooth row length; LTRL, lower tooth row length.

**TABLE 3:** Comparison of physiological indicators in *Eothenomys miletus.*

Trait	Regions					*F*	*P*
	DL	LJ	DQ	XGLL	JC		
Leptin levels	1.195 ± 0.022^a^	1.149 ± 0.170^a,b^	1.031 ± 0.022^c^	0.986 ± 0.027^c^	1.106 ± 0.026^b^	8.550	< 0.01
Food intake	7.490 ± 0.057^c^	7.812 ± 0.033^b^	8.602 ± 0.048^a^	8.652 ± 0.072^a^	7.786 ± 0.037^b^	55.125	< 0.01
RMR	2.098 ± 0.021^b^	2.138 ± 0.022^b^	2.474 ± 0.066^a^	2.587 ± 0.049^a^	2.146 ± 0.035^b^	16.099	< 0.01
NST	4.720 ± 0.031^b^	4.735 ± 0.032^b^	5.049 ± 0.045^a^	5.078 ± 0.048^a^	4.726 ± 0.039^b^	12.387	< 0.01
NPY	0.997 ± 0.021^b^	1.002 ± 0.030^b^	1.247 ± 0.033^a^	1.270 ± 0.030^a^	1.035 ± 0.023^b^	13.573	< 0.01
AgRP	0.974 ± 0.028^b^	1.015 ± 0.029^b^	1.189 ± 0.041^a^	1.204 ± 0.034^a^	1.020 ± 0.015^b^	6.700	< 0.01
POMC	0.982 ± 0.049	1.019 ± 0.104	1.029 ± 0.108	0.957 ± 0.102	1.001 ± 0.078	1.118	> 0.05
CART	1.001 ± 0.058	1.018 ± 0.089	0.977 ± 0.088	0.957 ± 0.093	1.027 ± 0.093	0.818	> 0.05

RMR, resting metabolic rate; NST, non-shivering thermogenesisg; NPY, neuropeptide Y; AgRP, agouti-related protein; POMC, proopiomelanocortin; CART, cocaine and amphetamine regulated transcription peptide.

**FIGURE 7:**
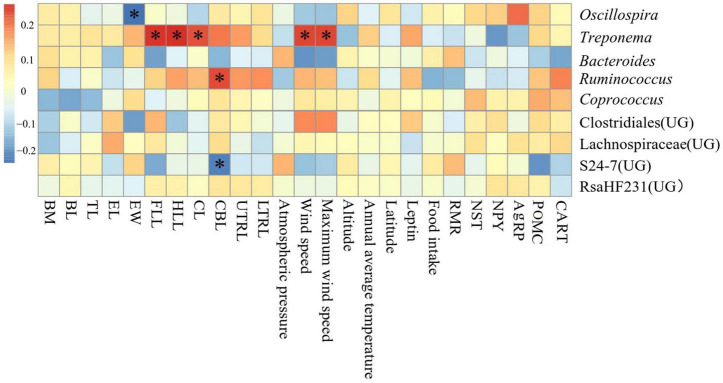
Heat map of environmental physicochemical properties in different regions related to the dominant microorganisms in *Eothenomys miletus.*

Correlation between environmental physicochemical properties in different areas and the dominant genus (top 9 in the relative abundance of all samples) in the feces was shown in Figure 7. Forelimb length, hindlimb length, cranial length, wind speed and maximum wind speed were significantly positively correlated with the abundance of *Treponema* (*P* < 0.05), ear width was significantly negatively correlated with the abundance of *Oscillospira* (*P* < 0.05), cranial base length was significantly positively correlated with the abundance of *Ruminococcus* (*P* < 0.05), and was significantly negatively correlated (*P* < 0.05) with abundance of S24-7(UG).

**FIGURE 8:**
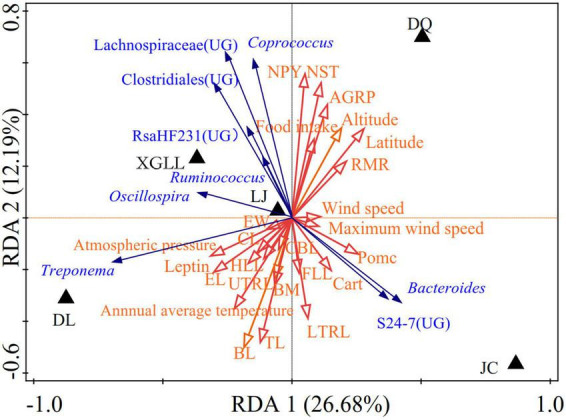
Redundancy analyses (RDA) of the correlation between environmental physicochemical properties and dominant microbial communities.

Correlation between environmental physical and chemical properties in different areas and the dominant genus was shown in Figure 8. Wind speed and maximum wind speed were positively correlated with the abundance of *Bacteroides* and S24-7(UG) in JC, RMR was negatively correlated with the abundance of *Treponema*, NST and NPY were negatively correlated with the abundance of *Bacteroides*.

### Co-occurrence network of microbe in the feces of *Eothenomys miletus* in different regions

**FIGURE 9:**
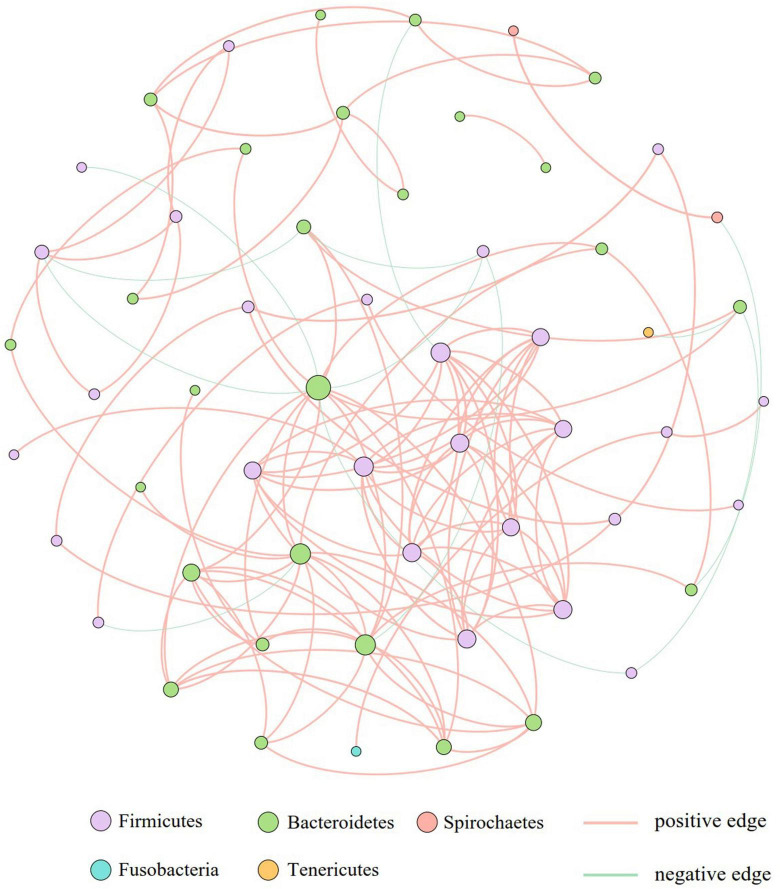
Dominant OTU co-occurrence networks in *Eothenomys miletus.*

Correlation of dominant OTUs in feces of *E. miletus* in different areas to describe the interaction between two microorganisms (OTU with *r* > 0.6, *P* < 0.05 and abundance > 0.001 for all samples) was shown in Figure 9 and Table 4. Spearman’s rank correlation between two microbes with *r* > 0.6, *P* < 0.05, suggesting their positive correlation (pink edges) and negative correlation (green edges) among microorganisms. It consists of 57 nodes and 122 edges (each node has an average of 2.140 edges). The average path length (APL) is 2.704, the average clustering coefficient (ACC) is 0.611, and the modularity index (MD) is 0.776 (value > 0.4 indicates that the network has a modularity structure). The nodes in the network were divided into five bacterial phyla, in which Bacteroidetes and Firmicutes were widely distributed, accounting for 93% of all the nodes.

**TABLE 4:** Topological properties of co-occurring networks.

	Gut
Nodes	57
Edges	122
Positive edges (%)	110 (90.16)
Negative edges (%)	12 (9.84)
Average degree	4.281
Density	0.076
Diameter	7
Average path length	2.704
Average clustering coefficient	0.611
Modularity	0.776

## Discussion

### Intestinal microbiota

Traditional separation method for identification of animals’ gut microbes is difficult to fully cover all types of bacteria, high-throughput sequencing technologies that cannot be cultured intestinal anaerobic bacteria detection, and the sequencing results with high accuracy can be complex reaction animal gut samples of bacteria community composition, abundance, structure characteristics and spatial distribution pattern (Woo et al., 2008; Xiaolong et al., 2017). Studies on intestinal microbiota in wild mice have found that environmental factors rather than host related factors play an important role in forming microbial community structure (Maurice et al., 2015). In previous studies, it was found that the intestinal microorganisms of *O. curzoniae* and *E. cansus* were mainly Firmicutes and Bacteroidetes at phylum level (Jing et al., 2018; Chuntao et al., 2019), which was similar to the results of the present study. At the phylum level, the dominant species of intestinal microbiota were the decomposing and utilizing plants. In our study, Firmicutes and Bacteroidetes were the dominant bacteria in the intestinal bacteria in *E. miletus*, and they accounted for more than 89% of the total intestinal bacteria. Studies have shown that Firmicutes and Bacteroidetes in the gastrointestinal tract are conducive to the digestion of cellulose and hemicellulose in food. It analyzed the microbial community in the rumen of *Cervus nippon* feeding on tussah leaves and found that 87.9% of 16S rRNA gene sequences belong to Bacteroidetes which can degrade fiber (Zhipeng et al., 2013). It also found that Firmicutes and Bacteroidetes in the rumen of beef cattle increased with the increase of dietary hay content, and this characteristic of Firmicutes was particularly prominent (Yanhong et al., 2011). At the genus level, the dominant genera of intestinal microbiota were S24-7(UG), Clostridiales (UG), and Lachnospiraceae (UG) in the current study. Among them, S24-7 belongs to Bacteroidetes, while Clostridiales and Lachnospiraceae belong to Firmicutes. Ormerod et al. (2016) found that a variety of bacteria in S24-7 could increase the abundance of enzymes that degrade carbohydrates, thereby improving the utilization of carbohydrates, such as starch in the nitrogenous extract of animal intestines. Clostridiales is beneficial to improve the digestion of cellulose and hemicellulose based plant food resources (Van Dyke and McCarthy, 2022). The main members of *Ruminococcus* are *Ruminococcusalbus* and *Ruminococcus flavefaciens*, they are the most studied strains in fiber degradation process and the dominant bacteria for cellulose decomposition (Koike and Kobayashi, 2001; Kohl et al., 2014). It is most important in the degradation of cellulose and hemicellulose, and its secreted cellulase is highly active. Therefore, it is not difficult to find that the dominant phylum and genus of intestinal microorganisms in *E. miletus* mainly focus on digestive fiber, indicating its high adaptability to phytophagous, which corresponds to the current results in *E. miletus* (Zwxun et al., 2000).

### Differences in intestinal microbiota in different regions

Based on the analysis of microbial α diversity, Chao1 analysis showed that the intestinal microbiota diversity in DL was significantly higher than that in LJ, DQ, and XGLL, while the intestinal microbiota diversity in JC was significantly lower than those in these three areas. However, Shannon diversity analysis showed that there was no significant difference in the evenness of intestinal bacterial flora among five regions, which may be attributed to the fact that DL is located in a relatively low latitude area, with relatively high winter temperature and relatively rich food sources. Therefore, the intestinal microbial diversity of *E. miletus* in this area was the highest. Although the dimension of JC is lower than that of LJ, XGLL, and DQ, the ambient temperature and precipitation in JC are relatively high in winter, so the plant growth may be rapid, *E. miletus* in JC need not be too far away from the nest to meet their needs in winter, which is also observed when we collect animal samples in their habitat environment. Therefore, the intestinal microbiota diversity was the lowest in JC. Due to the low temperature and poor food resources in winter in LJ, XGLL, and DQ, *E. miletus* in these three areas need to eat different kinds of food in winter, so the intestinal microbial diversity were in the middle.

Results of Venn diagram showed that the number of microbial genera in JC and DL was the highest, while that in LJ was the lowest, which also indicated that environmental differences would affect the diversity of intestinal microorganisms in *E. miletus*. XGLL and JC had the highest similarity of intestinal microbiota genera, reaching 65.16%, while LJ and DQ had the lowest similarity, only 58.88%, which may be related to the similarity of their food resource. It is not difficult to find that the differences among microbial genera were mainly concentrated in some genera of Firmicutes through the analysis of the differential microbial enrichment of *E. miletus* in different regions, and the reasons for the different enrichment were mainly due to the different food eaten by *E. miletus* in different regions. For example, studies on intestinal microorganisms of indoor and wild *O. curzoniae* showed that food and environment are the main reasons for the differences in intestinal microorganisms (Chunyan et al., 2021), which is consistent with the results of our study. Differences in food will lead to changes in gut microbial community structure (Ley et al., 2008), and differences in environmental microorganisms may also affect gut microbial community structure (Amato et al., 2013). The results of the present study showed that there was no significant difference in beta diversity based on the weighted UniFrac distance, indicating that the structure of intestinal microbiota was not affected by the region to a certain degree, which may be related to the feeding habits of *E. miletus*, and the food of this species is mainly plants with high fiber content. Of course, this point needs further study.

### Intestinal microorganisms and environmental factors, physiological and morphological indexes

A healthy gut microbiota is conducive to host metabolic homeostasis, and conversely, the composition of gut microbiota can be determined by ambient temperature and dietary energy (Dey et al., 2015). For mammals at high altitude, they usually have high Firmicutes/Bacteroidetes value (Daoliang et al., 2017; Guolei et al., 2019). This helps the host to mediate energy acquisition through the intestinal microbiota, and then helps plateau animals maintain metabolic balance and body temperature balance under low temperature conditions, which was consistent with the results of the current experiment. Since the living environment in *E. miletus* is about 1000–3000 m above sea level (Wanlong et al., 2009), in order to maintain the metabolic balance and body temperature, its NST and NPY were negatively correlated with the abundance of Bacteroides. *Ruminococcus* belongs to Firmicutes, which can produce short-chain fatty acid (SCFA) to enhance the protective function of intestinal barrier and reduce the colonization of opportunistic pathogens in intestinal tract (Zhaoqing et al., 2017). *Ruminococcus* plays an important role in cellulose degradation. Through microbial fermentation, cellulose can be converted into SCFA, which is an important energy source for epithelial cells and can provide about 10% of human energy (Yuhua et al., 2020). *Treponema* belongs to Spirochaetes, a genus that includes cellulose and xylan hydrolysis, which improves the ability to digest and extract valuable nutrients from fibrous natural plants (Debebe et al., 2017). It can produce SCFA, such as acetic acid and propionic acid, to provide energy for animals by degrading pectin in plant cell walls (Jing et al., 2013). There is a positive correlation between the microorganisms of these two genera and altitude in *E. miletus*, which also indicates that with the increase of altitude, the fiber content in the food gradually increases, so that the proportion of the microorganisms that decompose fiber in the intestine in *E. miletus* gradually increased, which is corresponding to the physiological data in this experiment. RMR and NST both in XGLL and DQ were significantly higher than that of the other three areas, and the altitude of these two areas is higher than 3300 m, which also shows that the *Bacteroides*, *Ruminococcus* and *Treponema* can affect the metabolism of the *E. miletus* and thus affect the change of its body mass. *Treponema* is also positively correlated with the forelimb length and hind length of the *E. miletus*, which determines the range of motion of *E. miletus* and the ability to avoid predators. The larger the range of motion may be related to the higher the amount of fiber in its food. Previous studies in our laboratory also found phenotypic differentiation in body indicators and skull morphology, and this phenotypic differentiation may be jointly affected by altitude, temperature or food (Yue et al., 2020). All the above results indicated that the physiology, morphology and intestinal microorganisms in *E. miletus* were all affected by environmental factors, making *E. miletus* more adaptable to the environment in different areas of Hengduan Mountain.

In conclusion, the present study was the first to compare the intestinal microbiota of *E. miletus* in different areas of Hengduan Mountain, which found that there were regional differences in intestinal microbiota of *E. miletus* in different areas, suggesting that the main reasons affecting the intestinal microbial community structure in *E. miletus* were the changes of temperature, altitude or food in different environment. Moreover, intestinal microbiota can affect body mass regulation by acting on physiological or morphological indexes in *E. miletus*.

## Data availability statement

The data presented in this study are deposited in the ENA repository, accession number PRJEB56142 (http://www.ebi.ac.uk/ena/data/view/PRJEB56142).

## Ethics statement

All animal procedures were within the rules of Animals Care and Use Committee of School of Life Sciences, Yunnan Normal University. This study was approved by the committee (13-0901-011).

## Author contributions

WZ conceived the study, participated in design, and coordination and drafted the manuscript. BY and TJ carried out the studies of body mass, gut microbiome, and metabolic rate studies were conducted. ZW carried out the morphological studies. All authors contributed to the article and approved the submitted version.

## Footnotes

1
http://data.cma.cn/

2
https://bioinfogp.cnb.csic.es/tools/venny/index.html
